# A Comparison of the Reliability of the Patellar Tendon-Trochlear Groove (PTTG) Distance and the Tibial Tuberosity-Trochlear Groove (TTTG) Distance Measured on MRI

**DOI:** 10.5704/MOJ.2003.006

**Published:** 2020-03

**Authors:** H Gupta, NS Batta, H Kataria, V Batra, AD Upadhyay, V Jain, P Mishra, N Goel

**Affiliations:** 1 Department of Sports Injury Centre, Vardhman Mahavir Medical College and Safdarjung Hospital, New Delhi, India; 2 Department of Radiodiagnosis, Mahajan Imaging, New Delhi, India; 3 Department of Biostatistics, All India Institute Of Medical Sciences, New Delhi, India

**Keywords:** PTTG distance, TTTG distance, patello-femoral instability, reliability study

## Abstract

**Introduction:**

An increased tibial tuberosity-trochlear groove (TTTG) distance is used for deciding a treatment plan in patello-femoral instability (PFI). The centre of the patellar tendon and the chondral trochlear groove can be directly visualised on MRI, and measured, giving the patellar tendon-trochlear groove (PTTG) distance. A study was designed to compare the inter-rater and the test-retest reliabilities of PTTG and TTTG measurements in MRI of patients without PFI and in a group with PFI.

**Materials and Methods:**

This cross-sectional reliability study was done on archival MRI films of 50 patients without patellar instability and 20 patients with patellar instability. TTTG and PTTG distances were independently measured by two orthopaedic surgeons and two radiologists. A hybrid PTTG measurement with bony landmarks on the femoral side and the patellar tendon landmark on the tibial side, was used to estimate the influence of the differences in the femoral and tibial landmarks on the difference in reliabilities. The intra-class correlation coefficient (ICC) was calculated for all four raters, as well as separately for each rater.

**Results:**

The PTTG distance had a higher inter-rater reliability (ICC=0.86, 95% CI=0.79-0.92) compared to the TTTG distance (ICC=0.70, 95% CI=0.59-0.80) in patients without PFI. Similar trends were seen in patients with PFI (0.83 vs 0.66). The inter-rater reliability for the hybrid PTTG distance was found to lie in between the TTTG and PTTG.

**Conclusions:**

The MRI-based PTTG distance had better inter-rater reliability compared with the MRI-based TTTG distance.

## Introduction

An increased tibial tuberosity- trochlear groove (TTTG) distance measured on CT scan is an important factor for patellar instability and is clinically used for defining the indications for the medialising tibial tubercle osteotomy (TTO)^1-3^. It approximates the amount of lateralisation of the patellar tendon insertion by using the apex of the tibial tuberosity as the landmark for the location of the patellar tendon. However, in comparison to a CT scan, the centre of the patellar tendon can be directly visualised on MRI and the patellar tendon- trochlear groove distance or PTTG distance measured. The chondral surface of the trochlear groove can also be easily demarcated in MRI and used in place of the underlying bony margins. Studies comparing TTTG and PTTG distances measured on MRI have found the patellar tendon to lie more lateral to the tibial tuberosity, resulting in larger PTTG distances^4, 5^. Thus, the two distances may not be used interchangeably.

Validity and reliability are two important qualities of a test^6^. Validity is the ability of the test to measure what it intends to measure^6, 7^. TTTG distance is a valid measurement for discriminating between patellar instability and control patients, the discrimination validity^8, 9^. While the validity of PTTG distance has not been assessed separately, theoretically, PTTG distance is a more appropriate and valid measure than TTTG distance, to measure the lateralisation of the patellar tendon insertion.

Reliability is a measure of the precision of a test, or its capacity to produce constantly similar results^6^. For two tests with similar validity and complexity, the one with a higher inter-rater and test-retest reliability would be preferred. Thus, in addition to being more appropriate, if PTTG distance is found to be more ‘reliable’ then this would certainly give it an edge over TTTG distance. The reliability of PTTG distance as compared to TTTG distance will be influenced by the ability of the raters to select the same landmark points repeatedly: the centre of the patellar tendon versus the tibial tuberosity on the tibial side, and the bony versus cartilaginous borders on the femoral side.

Although TTTG distance has been shown to have good reliability, the values in the literature range from 0.6 to 0.97^9-13^; while there is limited data in the literature on the reliability of PTTG measurements. One study reported good inter-rater and test-retest reliability of 0.91 and 0.96 respectively for MRI based TTTG, and an even better inter-rater and test-retest reliabilities of 0.98 and 0.97 respectively for MRI based PTTG distance^5^. However, another study, assessing CT and MRI based measurements of both TTTG and PTTG distances, reported slightly less inter-rater reliability of 0.82 for PTTG distance^9^.

The study was designed to compare the inter-rater and test-retest reliabilities of PTTG and TTTG measurements in MRI scans of patients, with no patellofemoral instability (PFI). A comparison was also done in MRI scans of a group of patients with PFI. In the study, a measurement with bony landmarks on the femoral side and the patellar tendon landmark on the tibial side, termed the hybrid PTTG distance, was also assessed to estimate the influence of the femoral and tibial landmarks on the difference in reliabilities with a hypothesis that the reliability for of the PTTG distance measurement was better than the TTTG distance measurement.

## Materials and Methods

It was a retrospective study conducted with archival MRI films from the radiology services located inside the medical centre, with films taken in January 2018 to June 2018. Ethical approval for the use of the archival data with a waiver of informed consent was obtained from the Institutional Ethics Committee, as part of a larger study. MRI scans on knee joints in patients aged 15 years to 60 years were included. The medical history of the patients was reviewed from MRI requisitions and reports. For the PFI group, MRI scans of patients with a clear history of patellar dislocation were included. Scans with doubtful recorded history or missing data were excluded from both groups. The exclusion criteria common to both groups were history or features suggestive of grade III injury of the major ligaments of the knee (ACL, PCL, MCL, LCL and PLC), previous knee surgeries, bipartite patella, grades III or IV cartilage lesions of the patella, grade III or IV osteoarthritis of the knee, fracture, metabolic disease, or tumour. Patients without PFI and not fulfilling any exclusion criteria were included in the non-PFI group.

For sample size calculation, the method described by Bujang *et al* for comparing intraclass correlation coefficient was used^14^. With the null hypothesis ICC (R0) as 0.80 and the minimum relevant difference in ICC as 0.1 (hypothesis ICC or R1 as 0.90), the number of raters (k)=4, alpha=0.05 and power (beta)= 0.80, the sample size was calculated using the formula:

N=1+[2(Zα + Zβ)^2^ k] / [(ln C0)^2^ (k-1)]

Where,

C0 = [1 + kθ0] / [1 + kθ1]

θ0 = R0 / [1-R0] ; θ1 = R1 / [1-R1]

N = 28.3

A higher sample size of 50 was used for the non-PFI group, with an equal number of males and females. For the PFI group, 20 MRI scans were available and were included.

The images were obtained in DICOM format and connotations related to patient identification and demographics were hidden. After the inclusion process, the personal details of the patients were not accessed, other than age and gender, to maintain patient confidentiality. The authors were blinded to the history and diagnosis and the measurement values of other raters or the rater’s previous measurements, at the time of taking the measurements, to mitigate potential sources of bias. All measurements were done by four raters, two orthopaedic surgeons and two radiologists. The orthopaedic surgeons were the raters 1, and 4, with a consultant with eight years of experience after specialisation and a registrar with three years of experience. The radiologists were the raters 2 and 3, and were consultants with 14 and 3 years of experience, respectively. Measurements were repeated by rater no. 1 for the PFI group and rater no. 4 for the non-PFI group after a gap of at least three weeks to assess test-retest reliability.

For measurements on the MRI images, the Jivex DICOM viewer was used by the two orthopaedic surgeons, and the GE MRI workstation was used by the two radiologists. All the measurements in the study were performed on Proton Density- Fat Saturated sequences. Distances lying on the lateral side of the trochlear groove were given a positive sign and measurements lying medial to it were given a negative sign. The method of measurement of TTTG and PTTG distances was as described in literature^5, 8, 10^

For measurement of TTTG distance, the axial images were scrolled from inferior to superior and the image with the deepest point of trochlea with a continuous ‘Roman arch’ was selected^10^. A line was drawn tangential to the bony margins of the posterior condyles of the femur. Another line at right angles to this line was drawn passing through the deepest portion of the bony trochlear groove. The images were then scrolled down and an axial cut with well-defined tibial tuberosity was identified. The perpendicular distance from the apex of the tibial tuberosity to the line passing through the trochlear groove was measured as the TTTG distance.

For measurement of PTTG distance, the landmarks used on the femoral side were the cartilaginous margins of the posterior condyles of the femur and the deepest portion of the cartilaginous trochlear groove. On the tibial side, the midpoint of the insertion of the patellar tendon was identified in the most proximal axial cut with the complete insertion of the patellar tendon. The perpendicular distance to this point from the line passing through the cartilaginous trochlear groove was measured as the PTTG distance.

The measurement of the hybrid PTTG distance, was done in much the same way as the TTTG distance, except that the midpoint of the patellar tendon insertion was chosen as described for the PTTG distance, instead of the apex of the tibial tuberosity.

“R Commander” was used for statistical analysis^15, 16^. The mean, range and standard error of the mean (SEM) were calculated for each of the three measurements for each rater separately. The difference between PTTG and TTTG distance was calculated for each measurement, and the statistical significance of the differences between PTTG and TTTG distances for each subject was assessed using paired t-test. The intra-class correlation coefficient (ICC) for inter-rater reliability along with the 95% confidence interval (95% CI) was calculated for all four raters, as well as for the two orthopaedic surgeons and two radiologist raters separately as a subgroup analysis. The two-way random effects model for the single rater was used for inter-rater reliability and ICC with a two-way mixed-effects model (single rater) was calculated for test-retest reliability, as recommended in literature^17-19^. The ICC values for PTTG and TTTG were compared. Also, the ICC values were transformed into z-statistic (Fisher z-transformation) and these z-values were then statistically compared for significant difference, with significance levels set at 0.05.

The reporting of the study conformed to the STROBE checklist for cross-sectional studies, as available on www.strobe-statement.org.

## Results

For including 50 scans of non-PFI patients with 25 males and 25 females, 137 scans were assessed for inclusion and 87 scans were excluded based on exclusion criteria or because of missing or doubtful recorded history. Scans of 20 PFI patients were available for the study. The measurements from all four raters were available for analysis for each of the 70 scans included in the study.

The mean age in the non-PFI group (n=50) was 28.5 ±1.5 years (range 15-56 years). There were equal males and females as per the selection process. The mean TTTG, PTTG and hybrid PTTG distances for the 50 patients for each of the raters along with standard error of mean (SEM) are given in (Table I). The values for all raters ranged from -2.2 to 15.1 for TTTG, -2.6 to 15.0 for hybrid PTTG and 0 to 15.0 for PTTG distance. The difference between PTTG and TTTG ranged from -4.4 to +6.4mm and was significant (p<0.05) for each rater. Overall, the average of PTTG values was higher than the average of TTTG values for each rater by 1.1 to 1.3mm.

**Table I:** Mean TTTG, hybrid PTTG and PPTG distances for the non-PFI group for each rater

	TTTG	Hybrid	PTTG	PTTG-TTTG difference
Rater 1	6.2±0.6	7.3±0.5	7.3±0.5	1.3±0.4
	(0.7 to 15.1)	(1.0 to 13.9)	(0.1 to 14.8)	(-4.4 to 6.3)
Rater 2	5.9±0.4	6.7±0.5	7.0±0.5	1.2±0.3
	(-1.8 to 13.8)	(-2.6 to 15.0)	(to 15)	(-4.4 to 6.4)
Rater 3	5.0±0.4	6.1±0.4	6.3±0.4	1.3±0.3
	(0.6 to 12.6)	(0.9 to 13.3)	(0.8 to 13.5)	(-2.5 to 5.1)
Rater 4(1)	5.8±0.5	6.6±0.5	7.0±0.5	1.2±0.2
	(-2.2 to 14.0)	(0.7 to 13.6)	(0.1 to 13.7)	(-2.2 to 3.8)
Rater 4(2)	5.7±0.5	6.5±0.5	6.9±0.5	1.2±0.2
	(-1.5 to 12.7)	(0.7 to 14.1)	(0.2 to 14.9)	(-3.7 to 4.0)

All values are given as Mean ± Standard Error of Mean (range). Rater 4(1) and Rater 4(2): two measurements by rater 4 for test-retest reliability

The ICC for inter-rater reliability for all 4 raters for PTTG distance was 0.86 (95% CI=0.79-0.92) (Table II), which was higher than the ICC for TTTG distance, 0.70 (95% CI=0.59-0.80). The corresponding z-values also had a significant difference (p=0.03). The ICC for hybrid PTTG distance (0.81) lay in-between the ICC values for TTTG and PTTG.

**Table II:** ICC for inter-rater and test-retest reliabilities for the non-PFI group

	ICC	95% CI	p-value 1	z value	Comparison groups	p-value 2
Inter-rater reliability, all raters						
TTTG	0.7	0.59 -0.80	<0.001	0.87	TTTG vs Hybrid	0.2
Hybrid	0.81	0.72 -0.88	<0.001	1.12	Hybrid vs PTTG	0.3
PTTG	0.86	0.79 -0.92	<0.001	1.31	TTTG vs PTTG	0.03
Inter-rater reliability, Ortho raters			<0.001			
TTTG	0.69	0.52 -0.81	<0.001	0.86	TTTG vs Hybrid	0.04
Hybrid	0.85	0.75 -0.91	<0.001	1.26	Hybrid vs PTTG	0.8
PTTG	0.86	0.77 -0.92	<0.001	1.29	TTTG vs PTTG	0.03
Inter-rater reliability, Radio raters			<0.001			
TTTG	0.73	0.53 -0.85	<0.001	0.93	TTTG vs Hybrid	0.1
Hybrid	0.84	0.72 -0.91	<0.001	1.21	Hybrid vs PTTG	0.2
PTTG	0.89	0.75 -0.95	<0.001	1.43	TTTG vs PTTG	0.01
test-retest reliability, rater no.			<0.001			
TTTG	0.88	0.81 -0.93	<0.001	1.39	TTTG vs Hybrid	0.2
Hybrid	0.93	0.88 -0.96	<0.001	1.66	Hybrid vs PTTG	0.8
PTTG	0.94	0.89 -0.96	<0.001	1.71	TTTG vs PTTG	0.1

ICC= Intra-class Correlation Coefficient; 95% CI= 95% Confidence Interval; p value 1= p value for the ICC for the null hypothesis of ICC=0; z value= Fisher-z transformation of ICC; p value 2= p value for comparison of the z-transformed ICC values for the comparison group mentioned; Ortho= orthopaedic surgeon; Radio= radiologist. Significance set at 0.05.

In subgroup analysis, the inter-rater reliability between the two orthopaedic surgeons as well as the two radiologists separately showed similar results, and the difference between the ICC values for TTTG and PTTG distance was significant (p<0.05).

The test-retest reliability, for rater no. 4, was much better for these measurements: the ICC for TTTG distance, hybrid PTTG distance and PTTG distance were 0.88, 0.93 and 0.94, respectively. The differences between these three values were not statistically significant.

The mean age in the PFI group (n=20) was 20.9 ± 0.8 years (range 15 to 27 years). There were eight males. The mean TTTG, PTTG and hybrid PTTG distances for the 20 patients for each of the raters along with standard error of mean (SEM) are given in (Table III). The difference between PTTG and TTTG ranged from -2.1 to +9.3mm. Overall, the average of PTTG values was higher than the average of TTTG values for each rater by 1.0 to 3.2mm.

**Table III:** Mean TTTG, hybrid PTTG and PPTG distances for the PFI group for each rater

	TTTG	Hybrid	PTTG	PTTG-TTTG difference
Rater 1(1)	9.7 ± 0.8	12.8 ± 0.7	12.8 ± 0.7	3.2 ± 0.5
	(2.4 to 15.8)	(5.7 to 18.2)	(6.5 to 18.3)	(-0.7 to 9.3)
Rater 2	12.1 ± 0.7	13.0 ± 0.6	13.1 ± 0.7	1.0 ± 0.5
	(3.0 to 16.2)	(8.6 to 19.7)	(6.6 to 19.7)	(-to 6.2)
Rater 3	10.3 ± 0.7	12.9 ± 0.7	12.4 ± 0.9	2.1 ± 0.6
	(4.9 to 16.5)	(6.6 to 19.4)	(4.5 to 19.1)	(-2.1 to 8.3)
Rater 4	11.4 ± 0.9	13.3 ± 0.8	13.4 ± 0.8	2.0 ± 0.4
	(0.4 to 16.6)	(3.9 to 18.9)	(to 18)	(-1.4 to 5.7)
Rater 1(2)	9.5 ± 0.7	12.4 ± 0.7	12.3 ± 0.7	2.8 ± 0.5
	(3.6 to 14.6)	(6.2 to 17.8)	(6.5 to 18.8)	(-1.4 to 8.8)

All values are given as Mean ± Standard Error of Mean (range). Rater 1(1) and Rater 1(2): two measurements by rater 1 for test-retest reliability.

The ICC values for inter-rater reliability for TTTG, hybrid PTTG and PTTG distances were 0.66, 0.80 and 0.83, respectively in this group (Table IV). These values were slightly lower as compared to the non-PFI group, but showed a similar trend, with the ICC value increasing from TTTG to hybrid PTTG to PTTG distance. However, the differences in corresponding z-values did not reach statistical significance. The inter-rater reliability for the two orthopaedic surgeons and the two radiologists separately also showed a similar pattern. Similar to the findings in the non-PFI group, the ICC for test-retestreliability for rater no. 1, were higher than the ICC values for inter-reliability for TTTG, hybrid PTTG and PTTG distances, 0.89, 0.91 and 0.914, respectively. All ICC values were significantly higher than zero (p=0.000).

**Table IV:** ICC for inter-rater and test-retest reliabilities for the PFI group

	ICC	95% CI	p value 1	z value	Comparison groups	p value 2
Inter-rater reliability, all 4 raters						
TTTG	0.66	0.44 - 0.83	<0.001	0.79	TTTG vs Hybrid	0.34
Hybrid	0.80	0.66 - 0.91	<0.001	1.11	Hybrid vs PTTG	0.79
PTTG	0.83	0.71 - 0.92	<0.001	1.20	TTTG vs PTTG	0.22
Inter-rater reliability, 2 Ortho raters			<0.001			
TTTG	0.68	0.27 - 0.87	<0.001	0.83	TTTG vs Hybrid	0.31
Hybrid	0.82	0.61 - 0.93	<0.001	1.17	Hybrid vs PTTG	0.67
PTTG	0.77	0.52 - 0.90	<0.001	1.03	TTTG vs PTTG	0.56
Inter-rater reliability, 2 Radio raters			<0.001			
TTTG	0.65	0.13 - 0.86	<0.001	0.77	TTTG vs Hybrid	0.29
Hybrid	0.81	0.57 - 0.92	<0.001	1.12	Hybrid vs PTTG	0.63
PTTG	0.86	0.67 - 0.94	<0.001	1.28	TTTG vs PTTG	0.13
test-retest reliability, rater 4			<0.001			
TTTG	0.89	0.74 - 0.95	<0.001	1.41	TTTG vs Hybrid	0.74
Hybrid	0.91	0.79 - 0.96	<0.001	1.52	Hybrid vs PTTG	0.93
PTTG	0.91	0.77 - 0.97	<0.001	1.55	TTTG vs PTTG	0.68

ICC= Intra-class Correlation Coefficient; 95% CI= 95% Confidence Interval; p value 1= p value for the ICC for the null hypothesis of ICC=0; z value= Fisher-z transformation of ICC; p value 2= p value for comparison of the z-transformed ICC values for the comparison group mentioned; Ortho= orthopaedic surgeon; Radio= radiologist. Significance set at 0.05.

## Discussion

The study found that the MRI based PTTG distance had higher inter-rater reliability, compared to the MRI based TTTG distance in patients without PFI. Similar trends were seen in patients with PFI. The test-retest reliability was quite similar for both PTTG and TTTG distances.

The TTTG distance is considered the gold standard measurement for assessing the lateralisation of the patellar tendon insertion and the majority of the available clinical data is on the TTTG distance^1, 2, 3, 5, 20^. However, the centre of the patellar tendon attachment is the point where the force of the patellar tendon acts. On the trochlear side also, it is the cartilaginous architecture of the trochlea that would better define the position of the patella and its tracking, rather than the underlying bony architecture^9, 21, 22^. The soft tissue landmarks of the patellar tendon and the deepest site of the cartilage in the trochlea, are the better anatomical and physiological landmarks compared with the bony landmarks, for an assessment of the lateralising vector force acting along the patellar tendon^4, 9, 10, 23^. Significant differences had been shown in different trochlear groove measurements when using cartilaginous compared to bony landmarks^24^. Similarly, the patellar tendon centre had been reported to have a more lateral placement as compared to the tibial tuberosity, resulting in a larger PTTG ^4,5^. In the present study, the difference between PTTG and TTTG values in the non-PFI group ranged from -4.4 to +6.4mm for all observers combined. In the PFI group, the differences between PTTG and TTTG distances were even higher, ranging from -2.1 to +9.3mm.

Studies reported good to very good intra-rater (ranging from 0.82 to 0.98) as well as inter-rater reliabilities for CT based TTTG measurements^9, 10, 11, 13^. However, some studies reported significant differences between the measurements taken by two raters, ranging from -13mm to +5mm^12, 25^.

There was limited data in the literature on the reliability of PTTG measurements. Pandit *et al*, reporting on normal values for MRI based PTTG distance in patients without PFI or ligamentous laxity, concluded that there was very good reliability in PTTG measurements^23^. However, this was based on the intra-observer coefficient of variation (CV%) of 9.04% and inter-observer CV% of 9.35%, and the ICC for assessing the reliability was not measured. Another study, assessing CT and MRI based measurements of TTTG and PTTG distances, described as functional TTTG, reported overall inter-rater reliability of 0.82 for all data^9^. However, separate reliability values for PTTG and TTTG distances were not reported.

Hinckel *et al* in their study comparing MRI based TTTG and PTTG distances, reported intra-rater reliabilities of 0.90 (95%CI= 0.831–0.942) and 0.93 (95%CI= 0.883–0.961) for TTTG and PTTG distances, respectively^10^. The inter-rater reliabilities were higher, at 0.97 and 0.98 for TTTG and PTTG distances, respectively^10^. Similar results were again reported from the same group comparing the MRI based TTTG and PTTG distances in 53 patients with patellar instability^4^.

Wilcox *et al* reported a significantly higher inter-rater reliability of 0.98 (95%CI= 0.968–0.983) for MRI based PTTG distance compared with TTTG distance (0.91; 95%CI= 0.811–0.953; p<0.001) for four raters^5^. For TTTG measurement, there were 44 instances where an observer’s reported measurement varied from the group mean by 2mm or more, compared with only three instances for PTTG measurement. The intra-rater reliability for PTTG was also shown by them to be significantly higher than for TTTG distance (0.972 for PTTG versus 0.961 for TTTG, p=0.009)^5^.

Thus, broadly, the inter-rater reliability for TTTG as well as PTTG distances were found in this study to be slightly lower than what was reported in the literature. In the non-PFI patients, the inter-rater reliability for PTTG was better than for TTTG for all four raters, and separately for each rater. A similar pattern was seen in ICC values in PFI patients. However, the test-retest reliability was good-to-excellent for both TTTG and PTTG. This could likely be because each rater had a particular way of defining “the apex of tibial tuberosity”, giving an excellent test-retest reliability but lower inter-rater reliability. The patellar tendon had well-defined boundaries and less variability was expected in determining its centre, thus giving excellent test-retest as well as inter-rater reliabilities. The inter-rater reliability for the hybrid PTTG distance was found to lie in between TTTG and PTTG, suggesting that the choice of landmarks on both the locations, distally on the tibia as well as proximally on the femur, could contribute towards better reliability.

The results of the present study are clinically relevant for the surgeons and radiologists since TTTG or PTTG distance was used for therapeutic decision making, that is, for defining the indications for a medialising tibial tubercle osteotomy (TTO)^1, 2, 3, 5, 20^. The results are generalisable since the raters included two radiologists and two orthopaedic surgeons with different levels of seniority, and a subgroup analyses of the radiologists and orthopaedic surgeons separately reported similar results. Although the TTTG distance was initially described on radiographs, CT was recommended for measuring this distance to increase the precision of measurement^8, 13^. This study showed that MRI provided a more direct measurement of the lateral deviation of the patellar tendon attachment with greater reliability, making PTTG even more suitable than the TTTG distance. The advantages of MRI over CT as a single radiological investigation in patients with patellar instability had been reported in the literature^9, 23^.

A limitation of the study was the small sample size for the PFI group and could be the reason why the difference in reliability of TTTG and PTTG using the z-transformation did not reach statistical significance in this group. However, Fisher z-transformation for statistical comparison of ICC is an approximate method, and a direct visual comparison of the ICC values was more relevant. Both the groups showed a similar pattern of reliability values, increasing from TTTG to hybrid PTTG and highest for PTTG.

Blinding of the raters in the study to the diagnosis as well as the measurement values of other raters or the rater’s previous measurements ensured the mitigation of any bias. For test-retest reliability, a gap of three weeks was kept, which was neither too short to result in memory bias nor too long. Further, as these were standard measurements and all raters were well versed with them, there was no risk of bias due to a learning curve.

## Conclusion

The MRI based PTTG distance had better inter-rater reliability compared with MRI based TTTG distance. Future studies should be aimed at defining treatment algorithms based on PTTG distance rather than TTTG distance.
